# White blood cells classification using multi-fold pre-processing and optimized CNN model

**DOI:** 10.1038/s41598-024-52880-0

**Published:** 2024-02-12

**Authors:** Oumaima Saidani, Muhammad Umer, Nazik Alturki, Amal Alshardan, Muniba Kiran, Shtwai Alsubai, Tai-Hoon Kim, Imran Ashraf

**Affiliations:** 1 Department of Information Systems, College of Computer and Information Sciences, Princess Nourah bint Abdulrahman University, P.O. Box 84428, 11671 Riyadh, Saudi Arabia; 2 Department of Computer Science and Information Technology, The Islamia University of Bahawalpur, Bahawalpur, 63100 Pakistan; 3 Department of Biotechnology, Virtual University of Pakistan, M.A. Jinnah Campus, Defence Road, Off Raiwind Road, Lahore, 54000 Pakistan; 4 Department of Computer Science, College of Computer Engineering and Sciences, Prince Sattam bin Abdulaziz University, P.O. Box 151, 11942 Al-Kharj, Saudi Arabia; 5 School of Electrical and Computer Engineering, Yeosu Campus, Chonnam National University, 50, Daehak-ro, Yeosu-si, Jeollanam-do 59626 Republic of Korea; 6 Information and Communication Engineering, Yeungnam University, Gyeongsan, 38541 Republic of Korea

**Keywords:** Computational biology and bioinformatics, Health care

## Abstract

White blood cells (WBCs) play a vital role in immune responses against infections and foreign agents. Different WBC types exist, and anomalies within them can indicate diseases like leukemia. Previous research suffers from limited accuracy and inflated performance due to the usage of less important features. Moreover, these studies often focus on fewer WBC types, exaggerating accuracy. This study addresses the crucial task of classifying WBC types using microscopic images. This study introduces a novel approach using extensive pre-processing with data augmentation techniques to produce a more significant feature set to achieve more promising results. The study conducts experiments employing both conventional deep learning and transfer learning models, comparing performance with state-of-the-art machine and deep learning models. Results reveal that a pre-processed feature set and convolutional neural network classifier achieves a significantly better accuracy of 0.99. The proposed method demonstrates superior accuracy and computational efficiency compared to existing state-of-the-art works.

## Introduction

Blood is composed of various components including different cell types and plasma. Blood performs the crucial function of transporting oxygen and nutrients to the body’s tissues and organs. Additionally, it helps eliminate waste products like carbon dioxide and ammonia. Blood performs several biological functions like transport of gases to and from the body and exchanging them in the lungs, clotting of blood by making oxyhemoglobin, immune response, and body temperature regularization. Blood serves several important biological functions including oxygen transport, cell regeneration, clotting, body temperature regulation, and immune response. It comprises four essential cellular components: red blood cells (RBCs), white blood cells (WBCs), platelets, and plasma. RBCs, which make up around 40% to 50% of total blood volume, are primarily responsible for the supply of oxygen throughout the body from the lungs where gaseous exchange takes place between the human body and its environment^1^. WBCs are found in both the lymphatic nodes and the blood. Although WBCs constitute only 1% of the blood in a healthy individual, they play a critical role in the immune system’s defense against foreign invaders^1^. WBCs actively seek out, identify, and bind to bacterial, fungal, or viral proteins to eliminate them and provide a first-hand defense against intruders. Several types of WBCs are identified each performing its specific function in our body’s immune response^2^.

WBCs also known as leukocytes, have the crucial function of providing immunity and the first defense wall in the human body against intruders and diseases. These cells can be categorized into four primary types: neutrophils, eosinophils, lymphocytes, and monocytes. Each type possesses distinct physical and functional characteristics^3^. Neutrophils are granulocytes equipped with enzymes that aid in the digestion of pathogens^4^. Monocytes are classified into macrophages that eat up the damaged cells including RBC platelets and detrimental invaders.^5–7^. Eosinophils are the defense force against viral infections and also contribute to inflation and tissue damage as observed in numerous diseases. Lymphocytes safeguard the body against tumor cells and cells infected by viruses^8,9^.

The WBC count holds significant importance in detecting diseases and predicting their prognosis, making it a crucial aspect of the healthcare industry. While manual methods are commonly used for determining these cell counts, labs can use alternative methods where automated apparatus is unavailable^10^. In the traditional differential method, blood samples are examined under a microscope to classify different types and count the WBCs by a pathologist manually^11^. Automated systems, on the other hand, utilize techniques such as cytochemical blood sample testing, Coulter counting, and static and dynamic light scattering to generate plots by analyzing the data, that represent distinct groups corresponding to different types of white blood cells^12–14^.

A complete blood count (CBC) is a blood test that provides valuable information about an individual’s health status. Traditionally, WBC classification has been performed by experienced medical personnel who visually differentiate WBCs based on their morphologies in blood smear samples observed under a microscope^15^. However, manual classification has several limitations and challenges. Human observation is prone to bias and may result in less accurate estimations. Additionally, manual classification is time-consuming, complex, and requires extensive expertise from inspectors, which may not meet the demands of precision and accuracy on a large scale in modern times. Consequently, there has been significant development in automatic WBC classification methods^16,17^. In cases of the presence of abnormal WBCs, manual results can be misleading, so the best option to avoid such misleading outcomes is automated methods. Automated processes are best suited to classifying and ascertaining the count of WBCs, providing improved accuracy and reliability.

**Figure 1:**
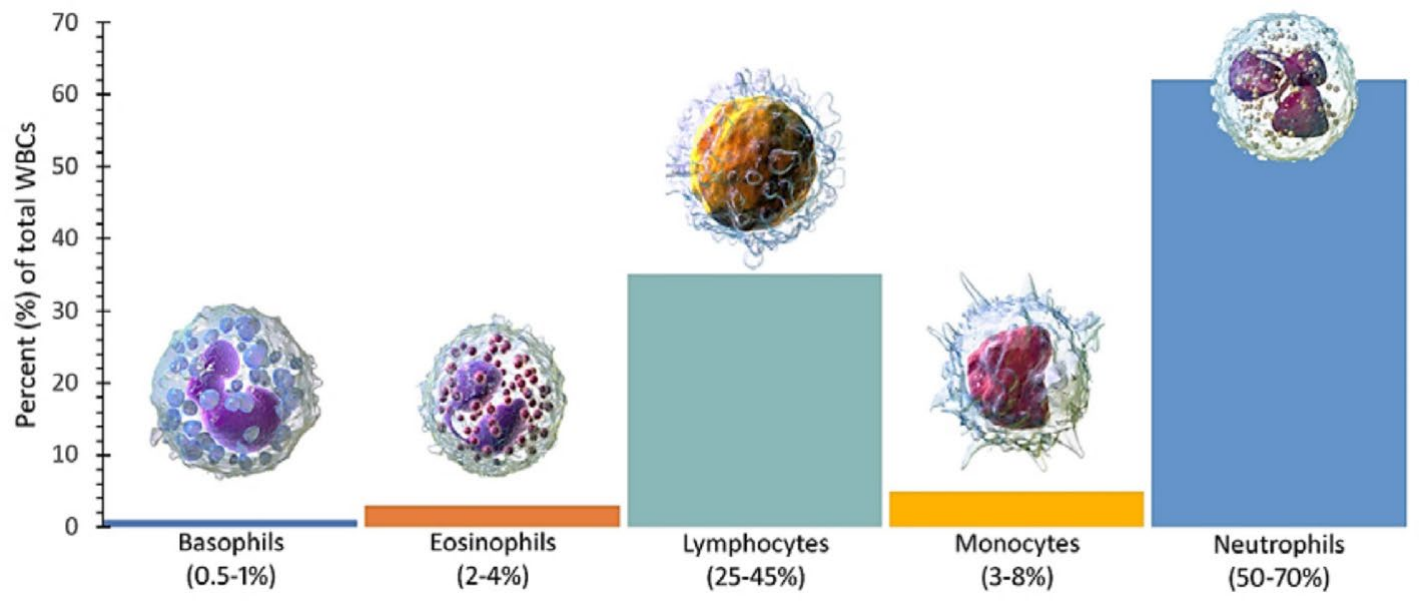
Average values for normal adult white blood cell count.

WBCs (leukocytes) are produced within the bone marrow and consist of nuclei and cytoplasm. There are five distinct groups of leukocytes: basophil, eosinophil, lymphocyte, monocyte, and neutrophil. These cells play a vital role in protecting the body by providing immunity to the body against infections, foreign substances, and microorganisms, forming a crucial component of the immune system. In a healthy adult human, there are typically around 4 to 11 billion white blood cells per liter of blood. This translates to approximately 7,000 to 25,000 cells per drop of blood. Figure 1 illustrates the average number of white blood cells in a healthy adult individual. Neutrophils are the most abundant type of leukocytes found in human blood. They have multi-lobed nuclei consisting of 3 to 5 lobes. Neutrophils account for 99% of all leukocytes, with polymorphonuclear cells representing approximately 70% of the total leukocyte count. Eosinophils exhibit a red color due to their uptake of eosin dye, a type of acid red dye, during staining. They possess large granules and have a lifespan of 1 to 2 weeks, comprising about 2 to 3% of all leukocytes. Eosinophils have an average diameter of 10 to 12 $\mu$m, and their nuclei are divided into two lobes.

Basophils are another group of leukocytes that have granules with a strong affinity for basic dyes, resulting in a deep blue-purple color. They possess an irregular nucleus composed of two lobes that are indistinguishable. Basophils are the least abundant type of leukocytes. Monocytes are the largest cells found in the peripheral blood, measuring approximately 15-22 $\mu$m in size. The nucleus of a monocyte exhibits folds and can have various shapes including round, lobular, kidney-shaped, bean-shaped, or horseshoe-shaped. Lymphocytes have the ability to undergo division and generate new lymphocytes. When they encounter antigenic stimulation, they undergo morphological transformation, differentiation, and multiplication. After neutrophils, lymphocytes are the most prevalent type of leukocytes in the blood.

Computer-aided diagnostic (CAD) methods and machine learning (ML) have extensively been used by several studies during the last two decades to address the limitations of WBC diagnosis and subgroup determination in laboratory image analysis. These studies have focused on analyzing blood smear images to diagnose, differentiate, and count various types of WBCs. ML, a prominent branch of artificial intelligence, encompasses algorithms and mathematical relationships that enable computers to learn from experience without explicit programming. The application of ML in medical data processing has yielded remarkable success, particularly in disease diagnosis^18^. In medical image processing, ML methods have proven beneficial in complex medical decision-making processes by extracting and analyzing image features. As the availability of medical diagnostic tools increased and generated large volumes of high-quality data, there emerged a pressing need for more advanced data analysis methods. Traditional approaches are unable to effectively analyze such vast amounts of data or identify underlying data patterns. In this context, the present study contributes in the following ways

- This study presents a framework for accurate white blood cell classification using an optimized convolutional neural network (CNN). Contradictory to existing works which predominantly rely on more complex transfer learning-based models, the deep learning CNN model is adopted to reduce computational complexity without affecting the model accuracy.
- For the most part, existing studies perform experiments on imbalanced datasets which might lead to model overfit for the majority class. Data augmentation is employed to balance the number of samples for various classes thereby reducing the probability of model overfitting.
- The efficacy of the proposed model is analyzed in comparison to several transfer learning models like ResNet, VGG16, MobileNet, and InceptionV3. In addition, the performance of the proposed model is compared with state-of-the-art approaches from existing literature.

The organization of this paper is as follows. “Section Related work” delves into the literature and background relevant to the WBC classification. The proposed framework, dataset, ML, and deep learning (DL) models are detailed in “Section Materials and methods”. Results are discussed in “Section Results and discussion” of this study. “Section Conclusion” concludes this study by outlining the avenues for future work.

## Related work

ML and DL have emerged as prominent research areas within the field of artificial intelligence. They have demonstrated promising outcomes in various applications including medical image classification, without the need for manual feature engineering. Umer et al.^19^ employed deep learning and ML techniques for medical image classification. He et al.^20^ focused on multi-label image classification using these methodologies. Rustam et al.^21^ explored text categorization, while Alturki et al.^22^ worked on brain tumor classification in medical imaging. Additionally, DL and ML have been applied to diabetes prognosis^23^, breast cancer detection^18^, and athlete gesture tracking^24^. In the context of WBC classification, numerous studies have been conducted. This section is about the discussion of some of the advanced WBC classification systems.

In order to achieve efficient classification of white blood cells, Elen and Turan^25^ introduced an automated ML system. Their experimentation involved utilizing geometric and statistical features, and the authors employed various ML models such as multiple linear regression (MLR), decision trees (DT), random forest (RF), Naive Bayes (NB), k-nearest neighbors (k-NN), and support vector machines (SVM). The results reveal that the MLR model achieves the highest accuracy of 95%. On the other hand, a WBC detection system based on DL is proposed by Praveen et al.^26^. The authors introduced you only look once (YOLOv3) object detection model for identifying and localizing WBC using bounding boxes. Additionally, the authors compared the results with the Faster R-CNN model utilizing the VGG16 architecture for the detection and classification task. The YOLOv3 model which incorporates contextual information about the available classes in a single pass, outperformed other models and achieved an accuracy of 99.2%.

Yao et al.^27^ proposed a highly efficient technique for WBC classification based on object detection. Their approach involved combining the segmentation and recognition of targets in a single step. They utilized two transfer learning models, namely Faster CNN and YOLOv4, for this study. The results demonstrated that Faster CNN achieved an accuracy score of 96.25%, which was 0.5% higher than YOLOv4. In another study, Rustam et al.^28^ employed RGB and texture features extracted from oversampled microscopic images for efficient WBC classification. They utilized the SMOTE (Synthetic Minority Over-sampling Technique) method for data oversampling. The Chi2 (Chi-squared) method was used for selecting RGB features. Along with machine learning models, deep learning models were also employed in their study. The results showed that the Random Forest (RF) model using RGB features yielded superior results, achieving an accuracy score of 97%.

Patil et al.^29^ tackled the challenge of classifying overlapping WBCs by utilizing the CNN model, recurrent neural networks (RNN), and a hybrid approach that combines both CNN and RNN. The authors employed the Canonical correlation analysis method and utilized the BCCD dataset. The proposed approach yielded an accuracy of 95%. Manthouri^30^ conducted a study focused on WBC detection using a hybrid approach that combines the CNN model with scale-invariant feature transform (SIFT). Feature detection was done using SIFT and the extracted features were used to train the CNN using the WBCs and LISC datasets. The suggested fusion prototype achieved impressive accuracy of 95.84% and 97.33% for the LISC and WBC datasets, respectively.

WBCs are automatically classified by an automated system based on DL in^31^. The authors experimented with different variants of CNN including DenseNet, VGG, SqueezeNet, and AlexNet. The results showed that DenseNet 161 achieved a perfect accuracy score of 100% on the BCCD dataset. Fatih Özyurt^32^ used a supervised ML approach for WBC detection, combining a CNN with an extreme learning machine (ELM) model. Several pre-trained CNN models, such as GoogleNet, AlexNet, VGG-16, and ResNet, were used for extracting the features. The extracted features were then utilized to train the ELM model, resulting in an accuracy of 96.03%. In another study^33^, the DenseNet121 model was utilized for WBC classification. Data normalization and data augmentation techniques were employed in conjunction with the enhanced DenseNet121 model. The recommended prototype attained an impressive accuracy score of 98.84% when evaluated on the KBC dataset.

Siddique et al.^34^ proposed an advanced DL system for WBC classification. The authors utilized the SqueezeNet model, a transfer learning-based approach, on the BCCD dataset. Experimental results demonstrated that the SqueezeNet model achieved an accuracy of 93.8%. In a study conducted by Girdhar et al.,^35^, a CNN-based approach was introduced for WBC classification. The researchers applied the proposed method to the WBC images dataset from Kaggle and accomplished an impressive accuracy score of 98.55%.

Table 1 performs a comparative analysis of the discussed research works. Most of these studies concentrated on classifying WBCs and employed transfer learning techniques, which typically require more computational resources compared to simple ML and DL models. It is important to highlight that some studies achieved impressive accuracy scores for WBC classification but they focused on a small number of WBC classes. Additionally, a few studies conducted experiments using imbalanced datasets, which can lead to overfitting in models. In contrast, this specific study tackles the issue of high computational costs by utilizing a simple DL model. While acknowledging the trade-offs associated with narrowing the scope of WBC classes, our study aimed to contribute a practical alternative to WBC classification methodologies, emphasizing efficiency without compromising essential diagnostic accuracy.

**Table 1:** Summary of the related work.

Ref.	Classifiers	Dataset	Achieved accuracy
^25^	DT, MLR, NB, k-NN, RFand SVM	350 blood smear images(self collected)	95% MLR
^26^	YOLOv3, faster RCNNusing VGG16	Kaggle (364 images)	99.2% YOLOv3
^27^	Faster RCNN,YOLOv4	Kaggle (364 immages)	96.25% Faster RCNN
^28^	DT, RF, SVM, k-NN, CNN,VGG16, ResNet15	IEEE dataport (3539images)	RF 97%
^29^	CNN, RNN, CNN+RNN	BCCD and Kaggle dataset	95.89% (CNN+RNN)
^30^	WTPSSR, SVM, DistanceClassifier, CNN	LISC and WBC(gitHub) datasets	LISC= 95.84% and WBC=97.33% using CNN with SIFT features
^31^	AlexNet, VGG Net 13, 11,ResNet 18, 34, 50, SqueezeNet 10, 11, DenseNet 121, 161.	BCCD dataset	100% DenseNet 161
^32^	CNN, ELM, AlexNet,GoogleNet, VGG-16, and ResNet,	BCCD dataset	96.03% using ELM
^33^	DenseNet 121 withdifferent patch sizes	KBC dataset	98.84% DenseNet 121
^34^	SqueezNet	BCCD	93.8%
^35^	CNN	Kaggle	98.55%

## Materials and methods

This section provides details about the dataset employed for WBC classification, as well as the steps taken for data pre-processing. Furthermore, it outlines the DL and ML models utilized for the classification task.

### Dataset

The dataset utilized in this study consists of five WBC classes including monocyte, neutrophil, eosinophil, lymphocyte, and basophil. The dataset is obtained from the IEEE Dataport^36^. Table 2 provides information regarding the number of images available for each WBC type. In total, the dataset comprises 3539 images which include 1464 augmented images, 667 raw images, and 1408 cropped and classified images.

**Table 2:** Dataset original and augmented record details.

WBC Type	Original	Augmented	Total
Neutrophil	319	194	513
Lymphocyte	905	–	905
Monocyte	82	418	500
Eosinophil	82	405	487
Basophil	20	447	467

### Data preprocessing

Several preparation techniques, including data augmentation and image resizing, are employed to yield optimal results and construct a robust image classifier. Image resizing, the process of adjusting the dimensions of an image file, involves either enlarging or reducing its size without eliminating any content. In this research, all images were resized to 256 $\times$ 256 before inputting them into the CNN for further processing.

Effectively training CNN models necessitates a substantial volume of training data to ensure the development of a well-performing model. Image augmentation is a commonly utilized method to enhance the efficacy of neural networks in achieving efficient image categorization through a relatively straightforward learning approach. The practice of altering existing images to generate additional data for model training is referred to as image augmentation. In this study, the ImageDataGenerator class is employed to create additional images^37,38^. Keras offers an image generator class that defines the configuration for image augmentation, encompassing features such as ’random rotation, shift, shear, and flips’, ’whitening’, and ’dimension reordering’, among others. Table 3 provides the parameter names and corresponding values utilized in the present study.

**Table 3:** Hyperparameters tunning with the ’ImageDataGenerator’ class for augmenting images.

Paramter	Value
rotation_ range	30
rotation_ range	30
width_ shift_ range	0.20
height_ shift_ range	0.20
shear_ range	0.25
zoom_ range	0.20
horizontal_ flip	True
fill_ mode	nearest

### Image preprocessing

**Figure 2:**
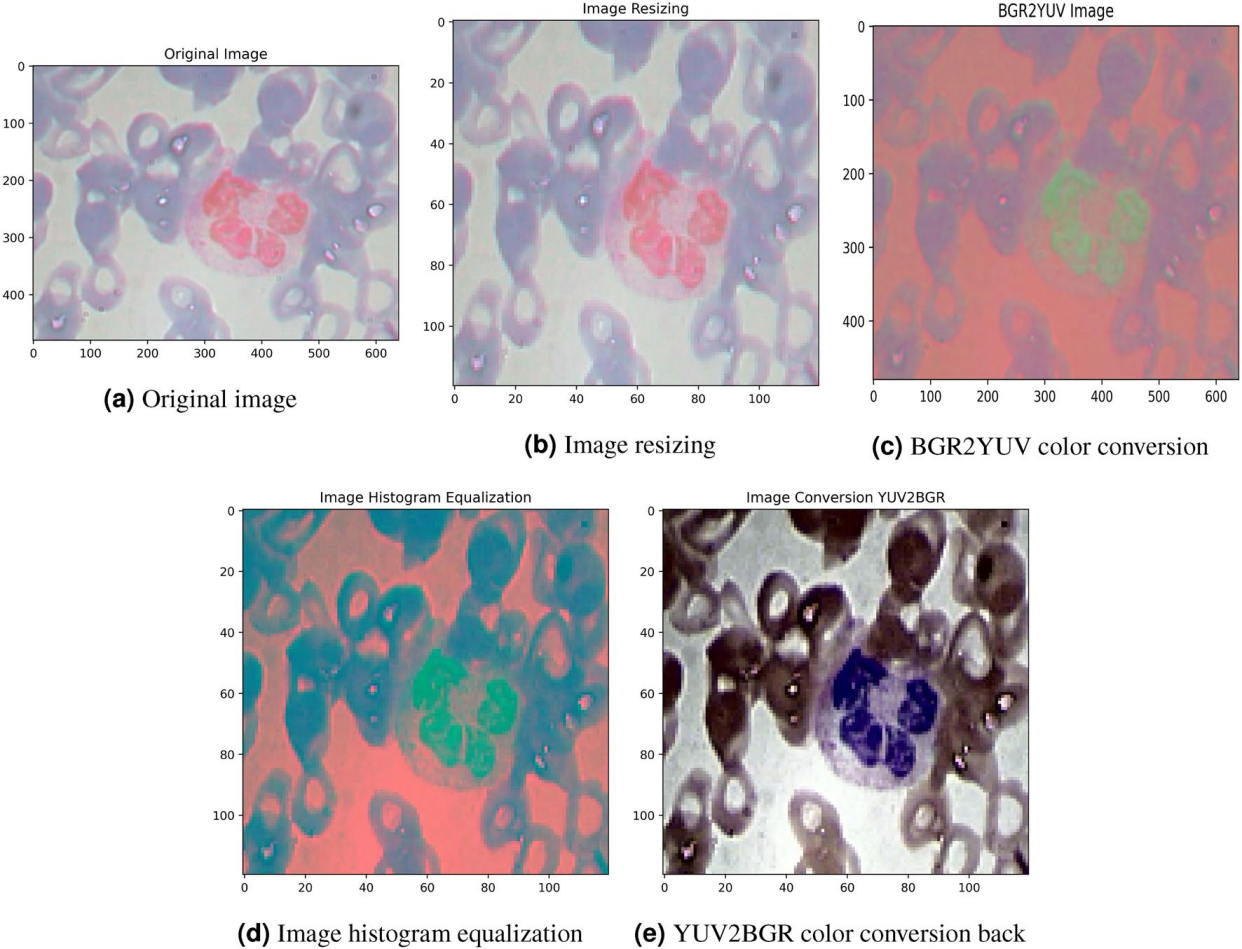
Complete data preprocessing procedure.

The preprocessing is carried out to eliminate noise from WBC images and enhance the training process. Initially, large input images were used leading to increased training time^39^. To address this issue, firstly, the image size of WBCs is reduced. The size of images may vary, as depicted in Figure 2. Initially, as shown in Figure 2b, the image size is adjusted to 120 $\times$ 120 $\times$ 3. For image edge detection, a value-based filter ([0, − 1, 0], [− 1, 6, − 1], [0, − 1, 0]) is applied to the images, with visible edges as an outcome as demonstrated in Figure 2c. The third step involves converting the BGR image to the luma component, red projection, and blue projection (YUV). This step results in reduced U and V channels’ resolution while retaining Y at full resolution. This conversion is done because luminance is preferred over color. By reducing the V and U channels, the CNN size can be significantly decreased. Figure 2d displays the outcomes of the BGR to YUV conversion. Finally, the YUV images are transformed back to BGR, incorporating histogram normalization and edge smoothing techniques. These steps contribute to the overall preprocessing pipeline described in the study.

### Deep learning models

The proposed system’s effectiveness is assessed by a combination of DL models, transfer learning models, and ML models. With the advancements in research, DL models and transfer learning models are widely utilized for WBC classification. Specifically, for WBC classification based on image data, CNN deep learning models and transfer learning models such as EfficientNet, U-Net, and MobileNet are commonly utilized. These models have shown promising results in accurately classifying WBCs.

Within the multidisciplinary realm of artificial intelligence, the DL model CNN stands out as an advanced architecture widely employed for a range of computer vision tasks. When compared to other network structures, CNNs have showcased superior performance and notable accomplishments in the field of computer vision^40^. CNNs possess a distinct characteristic known as invariance, enabling them to perceive images broadly. This means that even if an image contains dispersed facial attributes, CNNs can still recognize it as a person. In a CNN, convolution serves as a feature extraction technique, utilizing a specific-sized kernel. The kernel is thoroughly applied across the network with defined strides, determining the step size during the architecture’s implementation. The output obtained from this process is referred to as a feature map. Subsequently, the feature map’s size is reduced by deploying pooling. Each layer in the network consists of these aforementioned processes, and the network itself comprises multiple layers. Eventually, the image is flattened, leading to the formation of a partial or fully connected layer. Finally, a classification layer is utilized to categorize the image, determining its likelihood of belonging to any of the predefined classes^41^.

This paper explores various CNN architectures that have been tweaked to develop prototypes explicitly designed for WBC image classification. The aim is to create models that are optimized and customized to accurately classify WBC images.

### Transfer learning

In the transfer learning technique, a pre-trained prototype is used by the practitioners and the researchers for a new job. In the context of computer vision, transfer learning can be particularly advantageous as it leverages knowledge acquired from a previous task to enhance predictions for a new task. This approach is gaining increasing attention, primarily because it enables the training of deep networks even with limited input data. For transfer learning to be effective, the skills learned in the initial task should be applicable in a broader sense. Additionally, the input data for the new task must have the same dimensions as the data used to train the pre-existing model. In cases where the input size differs, a resizing operation is necessary to align the input with the pre-trained network before feeding it for further processing.

#### VGG16

It is a well-known variant of CNN, developed by the visual geometer group, and is known as VGG16^42^. The 16 in its name shows the 16 layers that have weights. It has convolutional layers of 3 x 3 filter with a stride value of 1 and has 2 x 2 layers of max pool and padding; these layers are followed by the softmax for the output. It is considered a large network because it has 138 million parameters. VGG16 is a pre-trained variant of a network trained on billions of images from the ImageNet database (Simonyan & Zisserman, 2014), more than thousands of classes can be classified with it.

#### InceptionV3

InceptionV3 is a deep learning model designed for image recognition tasks. It employs a unique architecture called the Inception module, which utilizes multiple convolutional filters of varying sizes to capture features at different scales^43^. This enables the model to learn hierarchical representations of images, detecting both low-level details and high-level patterns. InceptionV3 combines these multi-scale features through concatenation and learns global representations through auxiliary classifiers. It has been trained on large-scale image datasets and achieved state-of-the-art performance on various benchmarks. It is a powerful and efficient model that plays a vital role in computer vision applications, including object recognition and scene understanding.

#### ResNet50

ResNet50 is a popular deep learning model known for its impressive performance in image recognition and feature extraction tasks. It is a variant of the ResNet (Residual Network) architecture, which addresses the problem of vanishing gradients by introducing residual connections^44^. These connections enable the model to bypass certain layers and allow the direct flow of gradients during training, facilitating the training of very deep networks. ResNet50 consists of 50 layers and uses residual blocks as its building blocks. Each residual block contains multiple convolutional layers and shortcut connections. The model employs skip connections that add the output of a previous layer to a later layer, preserving and propagating important information across the network. By using residual connections, ResNet50 can effectively train deeper architectures while mitigating the degradation problem. It has been pre-trained on large-scale datasets, such as ImageNet, and has achieved state-of-the-art performance on various computer vision tasks. Due to its depth and skip connections, ResNet50 is capable of capturing intricate details and learning high-level representations, making it a valuable tool in applications like image classification, object detection, and semantic segmentation.

### MobileNetV2

MobileNetV2 is a compact architecture designed for mobile devices and embedded systems. It offers a smaller structure, reduced computation, and increased precision^45^. MobileNets influence deeply distinguishable convolutions and a couple of global hyper-parameters to balance the accuracy and efficiency. The basic concept of MobileNet revolves around disintegrating convolution kernels. By utilizing deeply distinguishable convolution, a standard convolution can be decomposed into a pointwise convolution and a deep convolution with a 1 $\times$ 1 convolution kernel. The deep convolution screens apply convolution independently to each channel, while the 1 $\times$ 1 convolution combines the productivities of the deep convolution layers. The N standard convolution kernel is substituted in this approach with M deep convolution kernels and N pointwise convolution kernels. While a standard convolutional filter combines inputs to produce new outputs, depthwise separable convolution divides inputs into two layers, one for filtering and the other for merging.

**Figure 3:**
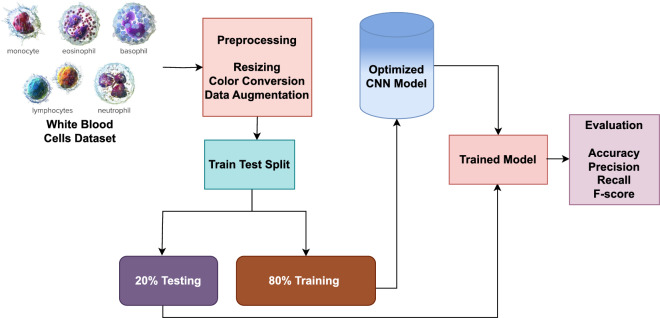
Proposed methodology diagram.

### Proposed CNN model

This study uses a customized CNN model, as shown in Figure 3. CNN is the best and the most widely used tool for computer vision projects. CNN consists of a large number of convolutional, pooling, and fully connected layers. These layers perform their functions and perform different tasks. For instance, a convolutional layer utilizes a fixed-size filter that is known as a kernel. This kernel is used for the extraction of the local features from the input image. Every time a new convolved image is obtained and after that, the conversation is applied to this image. This convolved image has a feature that was extracted from the image of the previous step. Considering *I*(*x*, *y*) is a two-dimensional image that is used as the input image and also considering *f*(*x*, *y*) is a two-dimensional kernel used for the conversations, then the conversation is captured by^41^.

1 $$\begin{aligned} y(i,j)=(I,f)(x,y)=\sum _{-\infty }^{\infty }\sum _{-\infty }^{\infty }I(x-u,y-v)f(u,v) \end{aligned}$$

When we use conversation, the pixel values at the edges cannot be ignored or will make use of the padding technique. The result of the convolution can be converted by utilizing non-linear activation.

2 $$\begin{aligned} sigmoid(x)=\frac{1}{1+e^{-x}} \end{aligned}$$

CNN not only contains a convolutional layer but it also contains fully connected and pooling layers. The pooling layers in the CNN are used for the summarization of local patches of convolutional layers. The pooling layers are also responsible for the calculations of the “ximum” and “verage” functions. These functions are also known as “ax pooling” and “average pooling”, with respect to the function they are performed. Pixel spacing is also a very important feature in the CNN model. In the CNN model pooling of every layer can be computed as

3 $$\begin{aligned} X_{ij}^{[l]}=\frac{1}{MN}\sum _{m}^{M}\sum _{n}^{N}X_{iM+m_jN+n}^{[l-1]} \end{aligned}$$

where *M* and *N* denotes the pooling space size and *I* and *j* shows the position of the output map.

For better classification of the CNN model dropout and dense layers are used. The extracted features are given to the fully connected layer. These layers have different weights with each association and need good computation resources. In this study, three layers are used with different filter sizes. The size of the filter is 32, 64, and 128. The purpose of using 32 filter sizes is to get minute information extracted from data. Then we use 64 and 128 filter sizes to broaden the information. The dropout value is set to 0.2. This layer is used to prevent the phenomena of overfitting. Then we use the flatten layer; it is used to convert the data into a single dimension because CNN is two-dimensional so, we use the flatten layer to convert the neuron into one dimension. After the flatten layer, a dense layer is used with 256 neurons, and after that dense layer of 4 neurons is used for target classes. For activation, softmax is used while the Adam optimizer is used for the optimization and the batch size is 32. Table 4 shows the details of the proposed CNN model.

**Table 4:** Architectural details of the proposed CNN model.

Name	Description
Convolution	Stride = (1 $\times$ 1), filter= (2 $\times$ 2, @ 256), kernel_regularizer=l2(0.01)
Dropout	0.5
Convolution	Stride = (1 $\times$ 1), filter= (3 $\times$ 3, @ 128), kernel_regularizer=l2(0.01)
Dropout	0.5
Convolution	Stride = (1 $\times$ 1), filter= (3 $\times$ 3, @ 64), kernel_regularizer=l2(0.01)
Dropout	0.5
Max polling	Stride = (2 $\times$ 2), pool size= (2 *times* 2)
BatchNormalization	–
Fully connected	Dense (120 neurons)
Dropout	0.5
Fully connected	Dense (60 neurons)
Dropout	0.5
Fully connected	Dense (10 neurons)
Layer	Flatten
Softmax	Softmax(4-class)

### Evaluation parameters

Accuracy alone does not provide a comprehensive measure of a classifier’s performance. Therefore, along with accuracy, other performance metrics such as precision, recall, and F1 score are computed. Accuracy represents the ratio of correct predictions to the total number of predictions. Precision, also known as the positive predictive value, quantifies the ratio of true positive predictions to the total number of positive predictions generated by a classifier for a particular class. Recall also referred to as sensitivity or true positive rate, measures the proportion of correctly predicted instances for a class out of all the actual samples belonging to that class. The F1 score is a statistical measure that combines precision and recall into a single metric for evaluating classifier performance. Every performance indicator has mathematical definitions presented below. Mathematical representations use FP (false positives), TP (true positives), FN (false negatives), and TN (true negatives).

4 $$\begin{aligned} Accuracy&= \frac{TP+TN}{TP+TN+FP+FN} \end{aligned}$$

5 $$\begin{aligned} Precision&= \frac{TP}{TP+FP} \end{aligned}$$

6 $$\begin{aligned} Recall&= \frac{TP}{TP+FN} \end{aligned}$$

7 $$\begin{aligned} F1 score &= 2 \times \frac{Precision\times Recall}{Precision+Recall} \end{aligned}$$

## Results and discussion

The objective of this study is to classify WBC images using various DL and ML models. The performance of different approaches, along with their computational complexity, has been analyzed through comparative analysis. In this section, the individual results of all models on the provided datasets under various scenarios are presented.

### Experimental setup

For the training of the models, this study uses a Dell PowerEdge T430 machine. The machine features a graphical processing unit of 8GB equipped with 2xIntel Xeon processors, each consisting of 8 cores running at 2.4 GHz. Additionally, it has 32 GB of DDR4 RAM. The training process required approximately 50 minutes to complete the learning phase and make predictions. The epochs are set to 15, the batch size is 256, early stopping patience value is 3. The loss is calculated as the ’categorical cross entropy’ optimizer is ’adam’, and the activation function is ’Relu’ in all layers.

The proposed model is tested along with the transfer learning models such as VGG16, InceptionV3, MobileNetV2, and ResNet50. For the experimentation, we used 70% of the dataset for the training of the learning models and 30% is used for validation purposes. The dataset used in this study consists of four target classes EOSINOPHIL (Class 0), LYMPHOCYTE (Class 1), MONOCYTE (Class 2), and NEUTROPHIL (Class 3).

### Results of transfer learning models

This study leverages several well-known transfer learning models such as VGG16, InceptionV3, MobileNetV2, and ResNet50 for WBC classification to carry out a performance analysis with the proposed approach. The purpose of using these models is to check the efficacy of the proposed system for the WBC classification. Class-wise results of the transfer learning models for the WBC classification are shown in Table 5.

**Table 5:** Results of transfer learning models.

Classifiers	Class	Accuracy	Precision	Recall	F1 Score
VGG16	EOSINOPHIL(Class 0)	0.9609	0.93	0.92	0.92
	LYMPHOCYTE (Class 1)		1.00	1.00	1.00
	MONOCYTE (Class 2)		1.00	1.00	1.00
	NEUTROPHIL (Class 3)		0.92	0.92	0.92
InceptionV3	EOSINOPHIL(Class 0)	0.9720	0.95	0.94	0.95
	LYMPHOCYTE (Class 1)		0.99	1.00	0.99
	MONOCYTE (Class 2)		1.00	0.99	1.00
	NEUTROPHIL (Class 3)		0.94	0.95	0.95
MobileNetV2	EOSINOPHIL(Class 0)	0.7847	0.73	0.66	0.69
	LYMPHOCYTE (Class 1)		0.76	0.95	0.84
	MONOCYTE (Class 2)		0.85	0.87	0.86
	NEUTROPHIL (Class 3)		0.79	0.64	0.71
ResNET50	EOSINOPHIL(Class 0)	0.7458	0.73	0.32	0.45
	LYMPHOCYTE (Class 1)		1.00	0.95	0.98
	MONOCYTE (Class 2)		1.00	0.75	0.86
	NEUTROPHIL (Class 3)		0.50	0.92	0.65

Results of the transfer learning models show that VGG 16 achieved an accuracy score of 0.9609, InceptionV3 achieved an accuracy score of 0.9720 and MobileNetV2 achieved an accuracy score of 0.7847. For WBC classification, ResNet50 is the lowest performer and achieved an accuracy score of 0.7458. The performance of InceptionV3 and MobileNetV2 is slightly different. Regarding the class-wise precision, recall, and F1 score, results for LYMPHOCYTE (Class 1) and MONOCYTE (Class 2) are better from all models except for MobileNetV2 which show low precision scores of 0.76 and 0.85, for these classes, respectively. VGG16 and InceptionV3 show excellent results for all classes in terms of precision, recall, and F1 score.

### Results of proposed approach

For the WBC classification, the proposed CNN is applied to the WBC dataset, the same dataset that is used for transfer learning models. Similarly, the training and test split ratio is also the same. Experimental results for the proposed CNN model are given in Table 6. Experimental results reveal that the proposed CNN model obtained an average accuracy score of 0.9986 which is better than all transfer learning models used in this study.

**Table 6:** Performance of the proposed CNN models.

Class	Accuracy	Precision	Recall	F1 Score
EOSINOPHIL (Class 0)	0.9986	0.98	0.98	0.98
LYMPHOCYTE (Class 1)		1.00	1.00	1.00
MONOCYTE (Class 2)		0.99	0.99	0.99
NEUTROPHIL (Class 3)		0.98	0.98	0.98

Results of the proposed CNN show that the proposed model achieved an accuracy score of 0.9986 and also achieved good values for other evaluation metrics such as precision, recall, and F1 score. For class 1 (LYMPHOCYTE) proposed CNN achieved a 1.0 score for all evaluation metrics. Similarly, for class 2 (MONOCYTE) it achieves 0.99 scores for the precision, recall, and F1 score. While the other classes (class 0 and class 3), it has 0.98 scores each for precision, recall, and F1 score. Results demonstrate the superior performance of the proposed model compared to transfer learning models.

### Comparison of transfer leaning models and proposed CNN model

If we compare the average results of the classifiers used in this study it shows that the proposed CNN achieved an accuracy score of 0.9989, as shown in Table 7. It is followed by the transfer learning models InceptionV3 and VGG16 with 0.9720 and 0.9609 accuracy scores, respectively. ResNet50 shows poor performance with an accuracy score of only 0.7458 which is the least among all employed models. The proposed CNN model achieved an average precision of 0.99 while average scores for recall and f1 score are also 0.99.

**Table 7:** Comparison results of models used in this study.

Classifier	Accuracy	Precision	Recall	F1 score
VGG16	0.9609	0.96	0.96	0.96
InceptionV3	0.9720	0.97	0.97	0.97
MobileNetV2	0.7847	0.78	0.78	0.78
ResNET50	0.7458	0.81	0.74	0.73
Proposed CNN	0.9986	0.99	0.99	0.99

**Figure 4:**
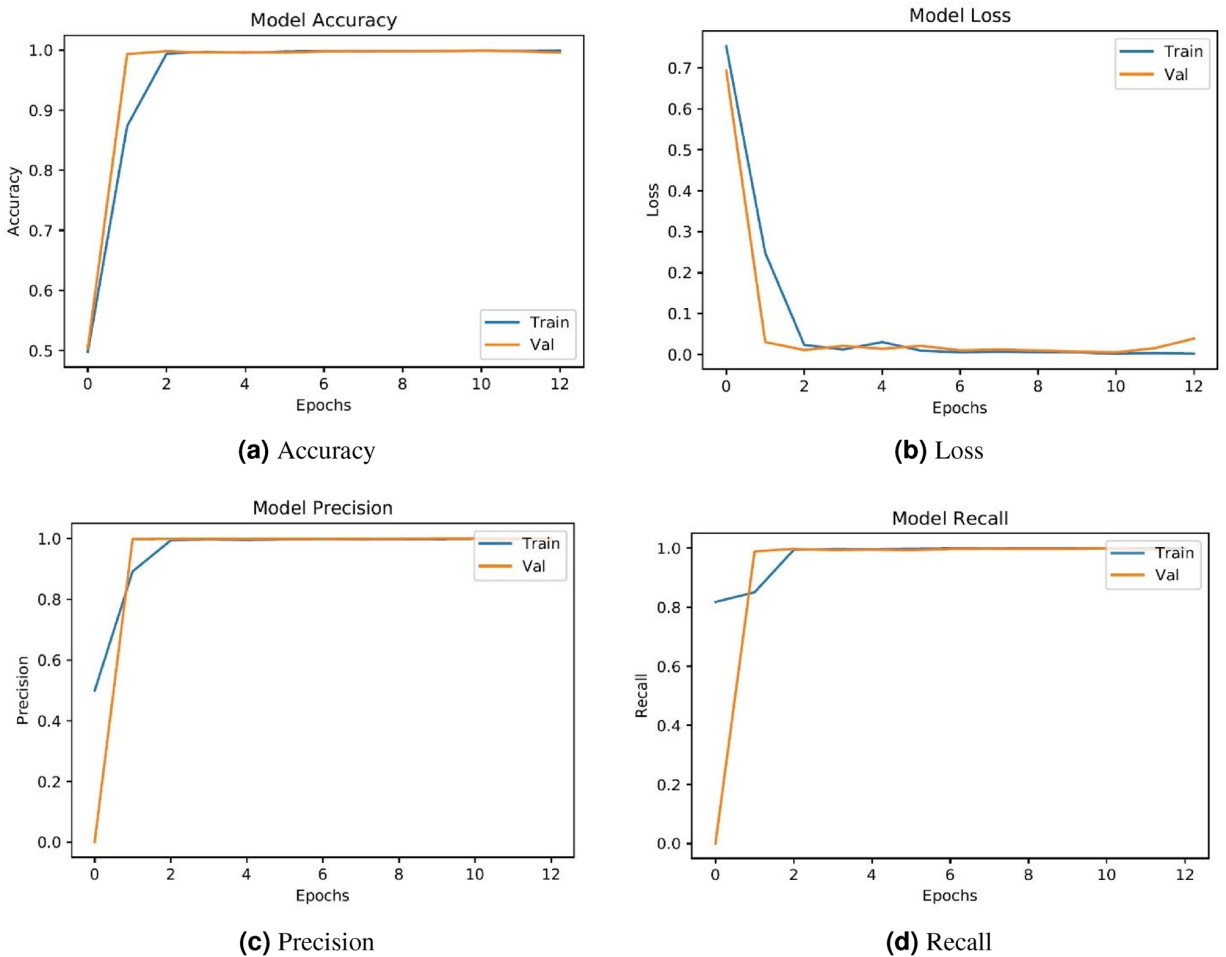
Training and validation accuracy and loss graphs for the proposed approach.

The proposed model training and testing graphs are given in Figure 4. Starting with epoch 1, it gradually improves the training and validation accuracy while the model loss is gradually reduced. An early stopping criteria helps to stop the model training when the performance is optimal. Graphs indicate that there is no overfitting occurring during these phases.

The hyperparameters details are shown in Table 4. The table also demonstrates the layers like dropout, batch normalization, and max pooling with supporting parameters like kernel, regularizer, etc. Using the dropout layer further helps to reduce the probability of model overfitting. Furthermore, before training, we added the parameter of early stopping that continuously monitors the training loss with a patience value of 3. If loss continues to drop consecutively 3 times then model training is stopped.

### Comparison of computational complexity

For a fair comparison regarding the computational complexity of the models, the execution time of all models is also analyzed in this study. Table 8 shows the executing time during training and testing of all employed models. Results indicate that the proposed model has the lowest time compared to other models. Transfer learning models have a higher number of trainable parameters which require a larger execution time. In comparison, the proposed CNN model has the least training and testing time.

**Table 8:** Training and testing time of all learning models in minutes.

Classifier	Training	Testing
VGG16	45.1	2.7
InceptionV3	49.3	2.3
MobileNetV2	55.0	1.9
ResNET50	51.7	2.2
Proposed CNN	39.7	1.3

### K-fold cross-validation

K-fold cross-validation is employed to ensure the robustness of the models. The results of 10-fold cross-validation are presented in Table 9, unequivocally showcasing that the proposed approach outperforms other models in terms of accuracy, precision, recall, and F1 score. Additionally, the proposed approach exhibits minimal standard deviation, emphasizing its reliability and consistency. These findings signify that the proposed approach consistently demonstrates strong performance across multiple folds, further instilling confidence in its reliability and robustness.

**Table 9:** 10-fold cross-validation result of the proposed model.

Model	Accuracy	Precision	Recall	F1 score
Fold-1	0.985	0.993	0.996	0.995
Fold-2	0.982	0.994	0.994	0.994
Fold-3	0.988	0.990	0.998	0.994
Fold-4	0.991	0.999	0.999	0.999
Fold-5	0.989	0.989	0.989	0.989
Fold-6	0.989	0.994	0.997	0.995
Fold-7	0.992	0.994	0.995	0.994
Fold-8	0.999	0.997	0.999	0.998
Fold-9	0.995	0.998	0.988	0.990
Fold-10	0.997	0.999	0.990	0.996
Average	0.994	0.994	0.991	0.992

### Ablation analysis

In order to check the participation of the pre-processing component, we performed an ablation study and considered the performance of all models with and without pre-processing phase. The results shown in Table 10 demonstrate that pre-processing plays an important role in improving the accuracy of all learning models.

**Table 10:** Ablation study to show the effect of each component of the proposed model.

Classifier	Accuracy with pre-processing	Accuracy without pre-processing
VGG16	0.9609	0.8434
InceptionV3	0.9720	0.8552
MobileNetV2	0.7847	0.7369
ResNET50	0.7458	0.7842
Proposed CNN	0.9986	0.9578

### Comparative performance analysis

A performance comparison is carried out to demonstrate the effectiveness and competitiveness of the introduced approach by comparing its performance with state-of-the-art methods for WBC diagnosis. In this regard, Table 11 shows the comparison of various existing approaches. In^25^, ML models were used for WBC classification, resulting in the highest accuracy of 95%. The study^28^ proposed a CAD system that utilized both ML and DL models for efficient WBC diagnosis. The study achieved the highest accuracy of 97% using the ML model RF. Similarly,^32^ obtained excellent results from various models including CNN, ELM, AlexNet, GoogleNet, VGG-16, and ResNet. Among them, ELM achieved a good accuracy score. The study^33^ employed DenseNet121 and achieved the highest accuracy of 98.84%. Along the same line,^34,35^ used single models and achieved an accuracy of 93.8% and 98.55%, respectively. Performance comparison with the above-mentioned state-of-the-art systems demonstrates that the proposed CNN model outperforms other models and achieves high accuracy for WBC detection, proving its effectiveness for WBC classification.

**Table 11:** Comparative performance analysis with state-of-the-art models.

Reference	Classifiers	Achieved accuracy
^25^	DT, MLR, NB, k-NN, RFand SVM	95% MLR
^26^	YOLOv3, faster RCNNusing VGG16	99.2% YOLOv3
^27^	FasterRCNN, YOLOv4	96.25% Faster RCNN
^28^	DT, RF, SVM, k-NN, CNN,VGG16, ResNet15	RF 97%
^29^	CNN, RNN, CNN+RNN	95.89% (CNN+RNN)
^30^	WTPSSR, SVM, DistanceClassifier, CNN	LISC= 95.84% and WBC=97.33% using CNN with SIFT features
^32^	CNN, ELM, AlexNet,GoogleNet, VGG-16, and ResNet,	96.03% using ELM
^33^	DenseNet 121 withdifferent patch sizes	98.84% DenseNet 121
^34^	SqueezNet	93.8%
^35^	CNN	98.55%
	Proposed CNN	99.86%

### Discussion

The proposed model (modified Novel CNN architecture) outperforms all other transfer learning models when its performance is compared in terms of accuracy, precision, recall, and f-score. Some of the reasons why transfer learning models cannot perform well are:

- Data Size and Distribution: In our case, the dataset distribution in each class is not very large. The dataset has a total of 1408 images belonging to different classes. With data augmentation, we tried to increase the records but still not sufficient. In these cases, training a model from scratch (modified CNN) might be more advantageous than using a pre-trained model. Transfer learning models often benefit from large and diverse datasets.
- Overfitting and Fine-Tuning: Transfer Learning, in some cases, especially with limited data, transfer learning models might suffer from overfitting during fine-tuning. If the transfer learning model is not fine-tuned carefully or if the task is substantially different from the pre-training task, it can lead to suboptimal performance.
- Dataset Quality and Labeling: If the quality of the dataset is high, and the labeling is accurate (as in our case), a model trained from scratch (modified CNN) can leverage this information effectively. Transfer learning models can be sensitive to noisy or mislabeled data.
- Architecture Design Choices: The choice of specific layers, activation functions, or other architectural elements in modified CNN could be well-suited to the nature of your task, contributing to better performance. In our case, we shared how we designed the novel modified CNN with different hyper-parameters and layers tuning 4.

### Validation using additional dataset

To validate the effectiveness of our proposed approach, we conducted additional experiments using a different dataset featuring blood smear images specifically for malaria parasite screening. Table 12 displays the outcomes achieved by applying the proposed approach to this dataset. Notably, the proposed method excelled in detecting malarial parasites within blood cell images, demonstrating an accuracy score of 0.9996, a precision score of 1.0, a recall value of 0.99, and an F1 Score of 0.99.

**Table 12:** Comparison results of models used in this study.

Classifier	Accuracy	Precision	Recall	F1 score
Proposed CNN	0.9996	1.0	0.99	0.99

## Conclusion

The significance of WBC classification emerges from their pivotal role in immune defense against infections and foreign agents. Detecting types of WBC types holds the potential to identify complications in the blood. However, prevailing research confronts limitations in accuracy, overfitting due to the usage of a large number of irrelevant features, and classification of a limited number of WBC types. To address these challenges, this study introduces an extensive pre-processing with a data augmentation phase to extract significant feature sets. This research work makes use of an optimized CNN model to ensure robust outcomes. Experimental findings underscore the efficacy of the approach attaining a remarkable 0.9986 accuracy score with the CNN model. The results are further compared with transfer learning models and state-of-the-art techniques to show the superiority of the proposed model. The proposed method demonstrates superior accuracy. As a future endeavor, we aim to explore further experiments employing machine-deep ensemble learning techniques.

## Data Availability

The dataset utilized in this research is publicly available and can also be requested from the author (Muhammad Umer).
